# XPS depth profiling of nano-layers by a novel trial-and-error evaluation procedure

**DOI:** 10.1038/s41598-024-69495-0

**Published:** 2024-08-09

**Authors:** Adel Sarolta Racz, Miklos Menyhard

**Affiliations:** Institute for Technical Physics and Materials Science, HUN-REN Centre for Energy Research, Konkoly Thege M. út 29-33, 1121 Budapest, Hungary

**Keywords:** XPS, Multilayer, Depth profile, TRIDYN, Tungsten–carbide, Materials science, Nanoscience and technology, Chemistry

## Abstract

In spite of its superior chemical sensitivity, XPS depth profiling is rarely used because of the alteration introduced by the sputter removal process and the resulting inhomogeneous in-depth concentration distribution. Moreover, the application of XPS becomes increasingly challenging in the case of the analysis of thin layers, if the thickness is in the range of 2–3 inelastic mean free paths (IMFP) of the photoelectrons. In this paper we will show that even in these unfavorable cases the XPS depth profiling is applicable. Herein the XPS depth profiling of a model system tungsten-carbide-rich nano-layer of high hardness and corrosion resistance is presented. We will show that the problems arising because of the relatively high IMFP can be corrected by introducing a layer model for the calculation of the observed XPS intensities, while the alteration, e.g. ion mixing, compound formation and similar artefact, introduced by the sputter removal process can be handled by TRIDYN simulation. The method presented here overcomes the limitation of XPS depth profiling.

## Introduction

The focus on nano-multilayers continuously increases both from pure material research and applied science point of views because of their widespread applications^1–3^. It is evident that the detailed study of such systems are of great importance. The characterizations of their structure and composition, due to the implicated size-range, is frequently carried out by various transmission electron microscopic techniques, which provide excellent structural information but somewhat limited information on the chemistry. To overcome the latter problem, surface sensitive analytical methods might be applied which provide together detailed in-depth concentration distribution and chemical information of the analyzed region. The most well-known surface sensitive methods are secondary ion mass spectrometry (SIMS), Auger-electron spectroscopy (AES) and X-ray photoelectron spectroscopy (XPS)^4–6^. The latter two techniques provide also information on the different bonding states present in the sample. The information depth of the AES is less than that the XPS thus its depth resolution is better, while for the chemical information the XPS is superior. If the thickness of the layers is in the range less than about 10–15 nm one of them or combinations of these methods provide the necessary information. It should be added that Angle Resolved XPS (ARXPS) method can be utilized to provide excellent chemical information and reasonable good depth resolution for multilayer system of thickness up to about 15 nm^7,8^. Having thicker multilayers one evident solution is applying sputter depth profiling to reveal the in-depth concentration distribution. However, the sputter removal process is obviously an invasive technique and introduces serious alterations to the sample that cannot be easily corrected during the routine evaluation of the depth profiles. In the case of AES depth profiling, by describing properly the sputter removal process, many of the technical problems have already been solved^9,10^ but it is evident that the chemical information it yields is strongly limited and its quantitative description contains difficulties (high background, backscattering factor etc.). XPS depth profiling is also widely used (see e.g. Li et al. review for functional layers^11^) provides more accurate description of the chemical composition but the invasive nature of the coupled sputter removal process and is even more disturbing and the higher mean free path of the ejected photoelectrons prevents reaching good depth resolution. There are works which offer at least partial solutions of these problems. Considering only the problem of the high inelastic mean free path of the photoelectrons e.g. Cao et al.^12^ proposed the deconvolution the depth profile. This method was further improved by Lubenchenko et al.^13^ consistently applying the Tougaard background^14^. The distortions due sputter removal can be handled by various ways. Using Ar gas cluster ion beam (Ar GCIB) for sputtering the damage introduced decreases as well as the sputtering yield and because of the latter it is limited only for soft organic materials. In the case of the most widely applied monoatomic (noble gas ion) sputtering based on AES depth profiling, one can define the less harmful conditions which are low ion energy, grazing angle of incidence and rotated specimen^9^. Cao et al.^12^ optimized the ion energy for low sputtering rate difference and ion mixing. Ignatova et al.^15^ XPS depth profiled relatively thick (~ 100 nm) oxide scale and found that the ion beam effects in general can be neglected but for the determination of the geometrical thickness they compared the results with TRIM simulation.

In this paper we will report on a new approach, a trial-and-error calculation, for the evaluation of XPS depth profiles where both the ion sputtering induced alterations and corrections for the high inelastic mean free path of the photoelectrons are considered; this method is similar to that which is frequently applied for the evaluation of the AES depth profiles^16^. This is a trial-and-error method where a trial in-depth concentration distribution is assumed. This structure is subjected to the actual ion bombardment in steps. The changes after a sputtering step are simulated by applying TRIDYN simulation. After each sputtering steps the XPS intensities are calculated by applying concentration dependent IMFP values. The test specimen is a C/W nano-multilayer (the individual layer thicknesses are between 9 and 25 nm) system. Previously we have shown that by irradiating this system by medium energy argon and xenon ions at room temperature tungsten carbide forms in the interface region exhibiting excellent corrosion and wear resistance features^17,18^. The in-depth concentration distributions before and after ion irradiation have been determined by AES depth profiling but the detailed chemical composition of the sample is missing.

This work will be divided into two parts. The first part is devoted to demonstration of the method. The XPS depth profiling of the non-irradiated, pristine C/W nano-multilayer sample of well-known structure. This part is used to describe the evaluation method which accounts for the high inelastic mean free path of the photoelectrons used by XPS and the unfavorable effects of low energy ion sputtering and verify its good performance. In the second part the described method will be used for determining the structure and chemistry of tungsten-carbide-rich films produced by medium-energy irradiation. This trial-and-error method which accounts both distortions (caused by the high IMFP and sputter removal introduced damage) as far as we know has not been applied for evaluation of XPS depth profiling. We emphasize that the evaluation method introduced here can also be applied on similar nano-multilayer systems thus, XPS depth profiling might be considered as a routine tool for determining the in-depth chemical composition of nano-layers.

## Experimental section

### Samples

The production of the samples have been described in reference^16^. Shortly a C/W multilayer structures were produced by sputter deposition (Balzers Sputron) from pyrolitic graphite and tungsten (99.99%) targets (diameter of 60 mm) on a single crystal silicon substrate, using plasma beam of 40 V/40 A. The targets were interchangeable in situ. The sputtering voltage was held constant at 1700 V with 0.6 A of the target current. The substrate temperature remained always below 100 °C during the deposition. The thickness of the sputtered layer was controlled by quartz-crystal microbalance. For the present studies a sample with structure—determined by cross-sectional transmission electron microscope (XTEM)^16,19^—of C 10.4 nm/W 24.5 nm/C 9.1 nm/SiO_2_ 2 nm/Si substrate has been used. For producing nano-layers of tungsten carbide rich regions the sample has been irradiated with various ions (Ar^+^, Xe^+^) in the medium energy range of 40–120 keV and the fluence was varied between 1 and 10 × 10^15^ ion/cm^2^^17,20^.

### XPS measurement and depth profiling

The XPS analysis was performed in an Escalab Xi^+^ instrument (Thermo Fisher Scientific). The spectra were recorded applying Al K α X-ray source (1486.6 eV). The size of the X-ray spot was 650 µm. No charge neutralizer was used. Tungsten (4f), silicon (2p), carbon (1s) and oxygen (1s) high-resolution spectra were measured within the spectral range of interest (ca. 20 eV around the core level emission peaks) at 20 eV pass energies with 0.1 eV steps and 50 ms dwell time per data point. The binding energy was set by fixing the C–(C,H) component of the C 1s peak at 284.8 eV^21^. All spectra were collected at normal emission angle. The amount of material producing the measured XPS spectrum is generally determined from the area of the XPS peak^22^. CasaXPS software^23^ was applied to determine this area from the high resolution spectra by applying Shirley-background subtraction. The spectra were decomposed to components by GL(30) peak shape (Gaussian–Lorentzian product function with a mixing parameter of 30) except the tungsten metallic component where Lorentzian Asymmetric Lineshape (LA) was used^24^. The peak area values are automatically corrected for the analyzer transmission function and escape depth (TPP-2M). These area values (CPSeV) are depicted in the figures showing the as measured profiles.

For depth profiling we have applied the default stetting of the equipment which is: angle of incidence of 45°, area of 2 × 2 mm and standing sample. Monoatomic Ar^+^ ion with an energy of 0.5 keV was used, the ion current was nominal 10 μA. The base pressure in the instrument was 8 × 10^–10^ mbar which was increased, because of the Ar admission, to 3.6 × 10^–8^ mbar during sputtering.

**Figure 1:**
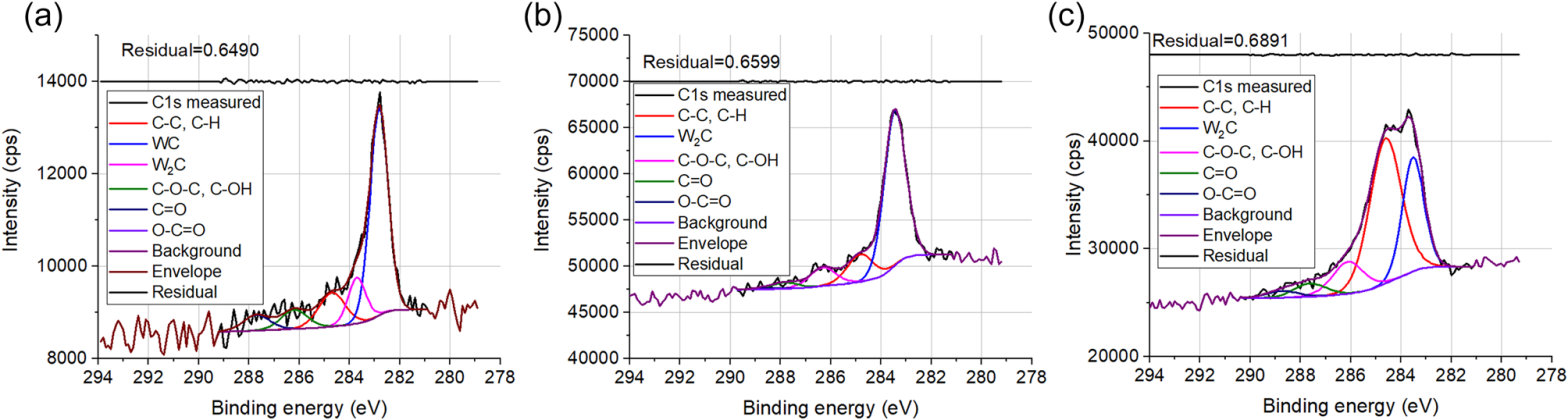
The as recorded C (1s) XP spectra of the (**a**) cermet, (**b**) 120 keV, 2.5 × 10^15^ Xe^+^/cm^2^ irradiated sample (carbide region) (**c**) 120 keV, 2.5 × 10^15^ Xe^+^/cm^2^ irradiated sample (transition region).

It is preferable to have reference spectra which helps in XPS evaluation^25^. For this purpose, spectra emitted from “real” carbide, a cermet (circular saw purchased from Mecut, Ceranisi, Italy) was measured, as well. Survey spectrum can be seen in Supporting Information Fig. S1. Figure 1a shows the high resolution C 1s XP spectrum measured in the cermet. The main peak is located at 282.8 eV (FWHM = 0.8 eV) which can be attributed to the W–C bond. However, we can see a small second peak located at 283.7 eV this can be assigned to W_2_C (tungsten-semicarbide)^26,27^. The other peaks located at higher binding energies can be attributed to the C–C, C–H bond (shifted to 284.8 eV) and C–O species (FWHM = 1.25 eV). Figure 1b,c presents present typical C 1s XP spectra obtained from different depth of our sample which experienced medium energy noble gas ion irradiation (120 keV, 2.5 × 10^15^ Xe^+^/cm^2^). Figure 1b depicts the carbide region where we can see that the main peak is located at 283.6 eV confirming the presence of W_2_C, with no other peaks located at lower binding energies. This indicates that the carbide produced in this depth region is (nearly exclusively) W_2_C. Figure 1c shows a transition region where graphitic C coexist with W_2_C. For the pristine and irradiated samples, the FWHM values varied slightly during depth profiling, the graphitic component was between 1.25 and 1.5 eV while the carbide component was between 0.85 and 1 eV.

The W4f line has a complex nature with many doublet subpeaks (see Supporting Information Fig. S2) causing uncertainty in the determination of the carbide content, therefore in the following only the C photoelectron line will be used for the characterization of the chemical composition of the sample. It is also noted that the IMFPs of the C photoelectrons are not affected by the slight change in the core electron energy that takes places upon compound formation. Additional high resolution C 1s and W 4f spectra for 6 consecutive sputtering steps for the pristine and 40 keV 1E16 Ar^+^/cm^2^ irradiated samples are provided in Figs. S3 and S4, respectively.

### Determination of the in-depth concentration distribution of the sample from the measured XPS depth profiles

#### “Routine” evaluation method

The usual evaluation protocol is based on the relative sensitivity method where the measured peak areas are scaled by the relative sensitivity factors (RSF) which are empirically derived from compounds of known composition^22^. This method assumes that the excited volume which produces the photoelectron peak is homogeneous.

#### Simulation of XPS intensities in case of inhomogeneous sample

If the concentration of the material is not homogeneous along the depth within the information depth, then the attenuations of the signals emitted from various depths are different and one should account for this. Tougaard in his seminal paper^28^ showed that the correct evaluation of the photoelectron spectrum (peak + background) contains information on the in-depth distribution as well and thus non-destructive depth profiling is possible. We should add that this method is also restricted to the information depth of the XPS, being 5–10 nm, and its application in the case of intermixed matrix is far not straightforward. Thus, in our case when samples with thicknesses of 20–60 nm are analyzed we cannot avoid the usage of destructive depth profiling.

A simple one dimensional procedure for the simulation of the photoelectron intensities has been used. Its essence is that the material is built up by layers and the intensities emitted by each single layers are summarized considering the electron transport in the matrix. The same approach had been successfully used for evaluating AES spectra^29,30^.

Thus, the sample has been built up by layers of equal thicknesses (1 nm) having various concentrations and densities; *c*_*ij*_ gives the concentration of element j in the layer i, which density is *Ni*. It is assumed that the exciting X-ray intensity is constant along the information depth (this is fulfilled since the information depth is lower than 10–15 nm) and thus the excitation is constant along the depth. Thus, the intensity of the photoelectrons of element j emitted from layer i is *I*_*ij*_ ~ *I* × *N*_*i*_ × *c*_*ij*_ × *σ*_*j*_ where *I* is the intensity of the exciting X ray and *σ*_*j*_ is differential cross-section of photoelectron production in the given circumstances. To get the emerging intensity of the photoelectrons emitted by element j the contributions of all layers should be summarized considering the attenuation during transport, which is given by the inelastic mean free path.

The transport of medium energy electrons in the solid has been studied thoroughly over the last 50 years^31,32^. In our evaluation of the electron transport we will use a simplified approach (because of the inhomogeneous matrix) which assumes that the current of the photoelectrons decreases exponentially while travelling in the solid. Their decay is determined by the inelastic mean free path (IMFP, λ) and the effect of elastic scattering^33^ is ignored. The IMFP values for pure matrices can be found in various sources; we will use the values given by the program QUASES-IMFP-TPP2M Ver. 3.0, which uses the TPP-2 M formula^34–36^. Table 1 summarizes some representative IMFP values used in our evaluations.

**Table 1:** Some of the IMFP values used in the calculation.

	W 4f in W	W 4f in C	C 1s in C	C 1s in W	Si 2p in C	O 1s in C
IMFP (nm)	2.0	3.8	3.3	1.7	3.7	1.8

As the material is built up by layers, the attenuation of the photoelectrons during crossing each 1 nm thick layers can be easily calculated by multiplying it by ${e}^{\frac{-1\, nm}{\lambda }}$^16^, where *λ* is the IMFP for the given photoelectron in the given layer considering the varying composition of the material. To get *λ* in a mixed matrix we will use the simple approach which tells that 1/*λ* = ∑*N*_*j*_*c*_*ij*_/*λ*_*j*_ where *λ*_*j*_ is the IMFP in pure *j* matrix.

The experimentally measured intensity is determined by the peak area of the emitted photoelectron. For the calculation of the peak area background subtraction is to be performed. For most practical work a fit-for purpose background is used, one has to choose between linear, Shirley and Tougaard background^37^. In our case the photoelectrons originating from various depths exhibit various backgrounds. We cannot account for these differences and as a rough approximation we will use always the commonly applied Shirley-background subtraction; the error due to this procedure was estimated to be 1–3%^38^.

#### Estimation of the sample alteration during sputter removal

First it should be mentioned that two of the parameters (angle of incidence and sample rotation) for ion sputtering applied are far from the so called “optimal” ones. In our previous work^16^ we have found, however, that for the given system the usual grazing angle of incidence ion sputtering is detrimental because of the large difference between the sputtering yields of the carbon and tungsten. We have found a minimum of the sputtering yield ratio around an angle of incidence of 65°. Since the optimum is not so sharp we chose the default setting of the equipment where the angle of incidence is 45°, which is not the best from the point of view material removal, but is optimal from the point of view of XPS analysis.

During sputter removal the reminding sample is altered due to the ion–solid interaction. Depending on the sputtering conditions the altered layer might be thinner/or thicker than of the information depth of the analysis. In the present case of 0.5 keV Ar^+^ bombardment, the thickness of the altered layer is less than the information depth of a typical photoelectron, meaning that the emitted photoelectrons originate partly from the altered layer and partly from the unaltered matrix, which should be accounted for in the procedure of determining the initial in-depth concentration distribution of the sample.

There is a huge literature in ion–solid interactions^39–42^. We will use a Monte Carlo based TRIDYN^43,44^ program (SRIM-2013.00) applying binary collision models, allowing dynamic target changes that describes the ion beam induced alterations, preferential sputtering, ion beam mixing and vacancy formation^45^. The sufficient statistical quality and precision of the simulation was provided by choosing as high number of pseudoparticles which resulted in a MAXCHA value (measure of the statistical quality) of less than 0.02. We have shown previously that the TRIDYN simulation (by properly modifying some of the default parameters of the code) describes quite well the sputter removal and intermixing processes in the case of C/W system in a wide ion energy range for Ar^+^ and Xe^+^ ions^16,20^. The surface binding energies (which determine the sputtering yield) of C and W have been fitted to get the experimentally measured sputtering yield.

This simulation accounts only for ion mixing, surface enrichment etc. but not for compound formation, which however actually takes place in the present case. The process can sufficiently be described by a simple model which states that all minority W or C components in C and W matrix, respectively form compounds^20^. The compound formed was confirmed, based on the XPS results, to be W_2_C. Thus, for describing the results it will be assumed that both intermixing and compound formation occurs during sputter removal process.

#### Simulation of the concentration distribution which results in the measured XPS depth profile

Because of the various difficult alteration processes, no generally applicable analytical solution exists for deriving the initial structure from the measured depth profile. On the other hand, one can simulate considering chapter 2.3.2 and 2.3.3 the depth profile for any initial structure. The simulated profile can be compared with the measured depth profile and the initial structure is to be modified until reasonable agreement is found. This trial and error methodology provides the initial structure for any measured depth profile. A similar approach was already successfully applied in case of AES depth profiling^16^, as well.

**Figure 2:**
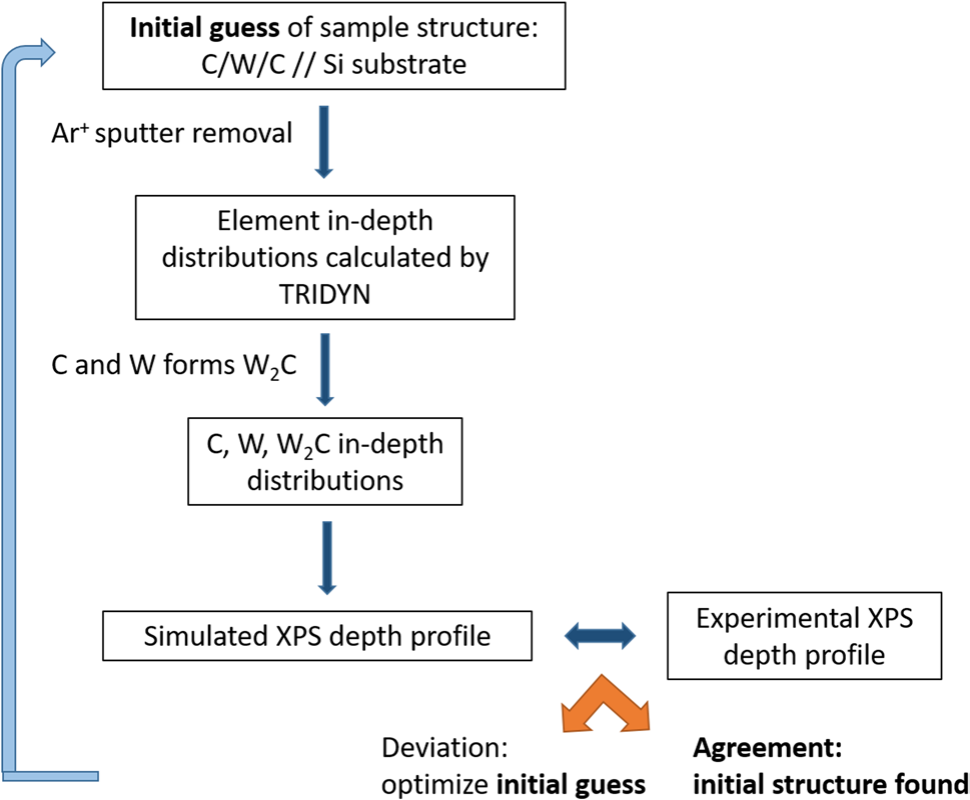
The proposed workflow for determining the initial concentration in-depth distribution of thin layer samples based on the measured XPS depth profiles.

Two ways of simulation will be applied. a. For demonstrating the effect of the relatively high IMFP of the photoelectrons the so called peeling procedure will be used. In this case we simulate a “damage free” depth profile by simply peeling the sample. Peeling means that thin layers (1 nm) of the material are removed sequentially from the arbitrarily built test sample and after every removal steps the XPS signals are calculated. In this simulation one cannot account for any ion bombardment effects; e.g. the imbedding of the bombarding Ar atoms is neglected. b. The sputter removal process is applied on the arbitrarily built test sample by means of the TRIDYN simulation, and after each sputtering steps (by removing thickness of about 0.5–1 nm) the photoelectron intensities are calculated producing the simulated depth profile. For better understanding Fig. 2 shows the proposed workflow for determining the initial in-depth concentration distributions of the thin layer sample producing the experimentally measured XPS depth profiles.

## Results and discussion

### Simulation of the XPS depth profile of pristine sample

The structure of the pristine sample has been determined previously^16,19^. It is: 10.5 nm C/24.5 nm W/9 nm C/2 nm SiO_2_/Si substrate. The C/W and W/C interfaces are sharp and clean (no intermixing, adlayer etc. are found) and the layers are amorphous, while the Si substrate is crystalline^16,19^.

**Figure 3:**
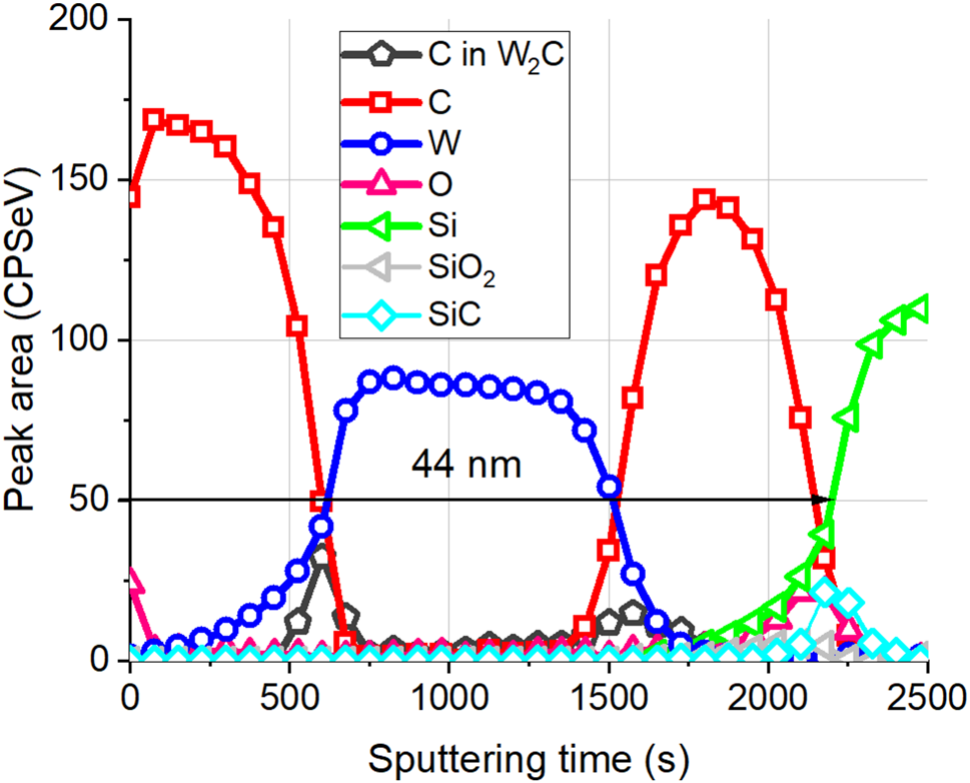
The as measured XPS depth profiles obtained on a pristine sample with structure of 10.5 nm C/24.5 nm W/9.0 nm C/2 nm SiO_2_/Si substrate. W, Si and C sign the XP intensities emitted from elemental W, Si, and graphite, while C in W_2_C stands for the C line which is emitted from W_2_C compound. SiO_2_ and SiC sign the photoelectron intensities of Si emitted from SiO_2_ and SiC compounds.

First we carried out the simulation of the XPS depth profile of this sample with well-known structure to validate our proposed protocol, and to show the relative weight of the various distortions that might appear during XPS depth profiling. The as recorded XPS depth profile obtained on the pristine sample is shown in Fig. 3. The conditions of the ion bombardment used for the depth profiling were: 500 eV Ar^+^ ions with angle of incidence of 45°, and the sample was not rotated during the ion sputtering. The XPS peaks have been identified by means of their binding energies.

**Figure 4:**
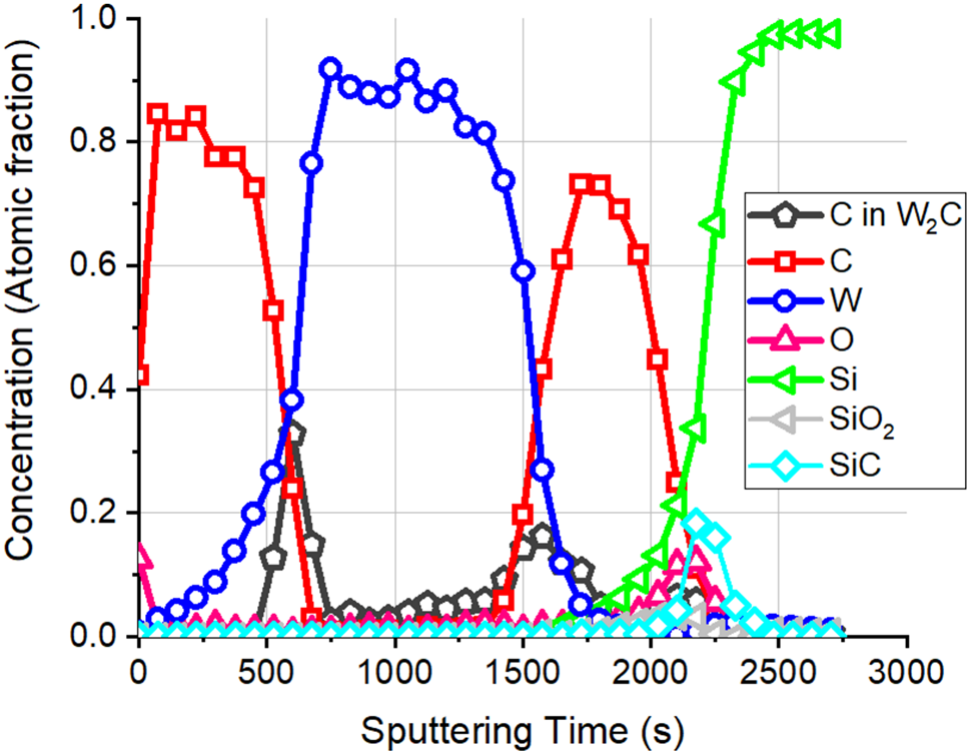
The result of a routine evaluation^22^ of the XPS depth profile shown in Fig. 3.

At first glimpse the result is rather disappointing; the as recorded XPS depth profile show strongly intermixed layers separated by wide interfaces and demonstrates the presence of a new components (expected based on Ref.^13^) of W_2_C and SiC, thus its resemblance to the initial (known) structure is very moderate. First we checked the applicability of the routine evaluation procedure^22^; the result is shown in Fig. 4.

According to this evaluation the sample (initial condition) depth profiled contains strongly intermixed carbon and tungsten regions which are connected by wide transitions. Besides the elemental components tungsten carbide (W_2_C) and silicon carbide (SiC) also appear. The proposed structure is gravely different from the initial structure of the sample, thus, the routine evaluation process for layer system having thickness comparable with those of the actual IMFPs cannot be used.

In the proposed evaluation procedure, we considered that the as recorded XPS depth profile showed strong intermixing, wide interfaces, appearance of new compound which might arise partly because of the sputter removal process applied during depth profiling and partly because of the non-negligible IMFP of the analyzed photoelectrons. The two processes can be evaluated independently.

First we consider the distortion of XPS depth profile only due to the finite IMFP of the photoelectrons, by simulating a “damage free” depth profile by simply peeling the initial sample.

**Figure 5:**
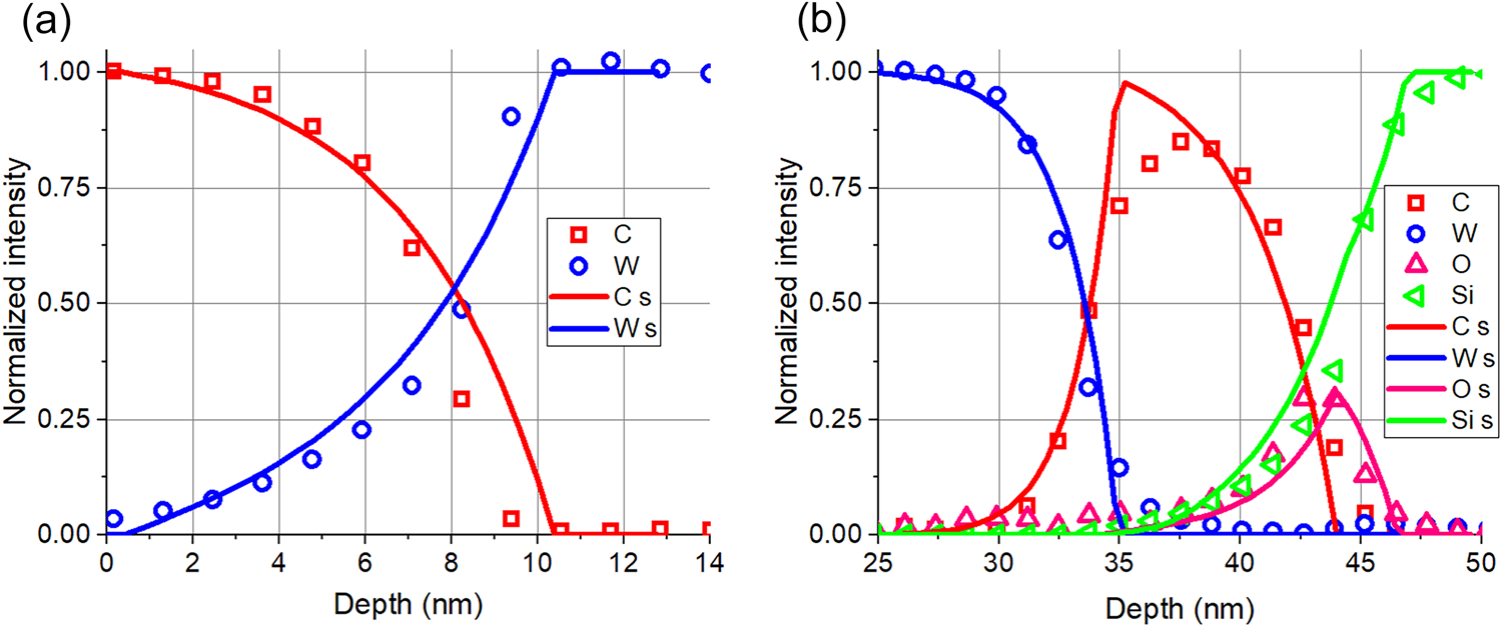
The comparison of the as measured depth profiles (symbols) with that of simulated ones (lines) in the case of peeling in the range of (**a**) C/W interface, (**b**) W/C; C/SiO_2_ and Si interfaces.

The comparison of the as measured depth profile with that simulated during the peeling process is far for being straightforward. The problem is that during the measurement the XPS intensities are measured as a function of sputtering time (s), while in the case of peeling given thickness of layer is removed that is the XPS intensities are calculated as a function of removed thickness which is regularly called as depth (nm). If the sputtering yield is known the sputtering time can be converted to number of atoms from this, if the density is also known, the removed thickness can be calculated. Since the sputtering yields of C, W and Si are rather different being 0.83, 1.05 and 1.36 (using Ar^+^, 0.5 keV, and 45° angle of incidence) the conversion of sputtering time to depth might result in errors. For this reason, only the interface regions where the horizontal axes can be fitted with reasonable accuracy are shown in Fig. 5, instead of the entire profile.

The agreement between the simulated depth profiles of the main components, obtained in the case of peeling, and that of the measured depth profiles is promising. The deviations between the corresponding curves are focused to the narrow region of the interface. That is, simply the relatively high IMFPs of the photoelectrons result in the features which seem to be intermixing. Note that the “intermixing” is much larger at the C/W interface than that at the W/C interface. This can also be easily explained; the IMFP of the photoelectrons being 3.8 nm W 4f in C and 1.7 C1s in W, respectively. Consequently, the “penetration” of W to C (C/W interface) is much higher than the “penetration” of C to W (W/C interface). Thus, the correction for IMFP is absolutely important.

A C/W/C/SiO_2_/Si system was peeled thus W_2_C (which appeared in the measured spectrum) evidently did not appear. To understand its appearance first of all let emphasize that the energies of W photoelectrons emitted from pure W and W_2_C are different, thus we can measure them separately but their IMFPs are the same. The very same is true for the C as well. It follows if the W_2_C is present in the sample the shape of the C in W_2_C profile should be similar to that of metallic W (the IMFP values are rather close). Considering Fig. 3 it is not the case; the W and C in W_2_C profiles in the first C layer are broad and sharp, respectively. It means that the W_2_C was produced during the sputter removal process. We note that this observation agrees with the XTEM results which did not show W_2_C layer on the interface^16,19^.

**Figure 6:**
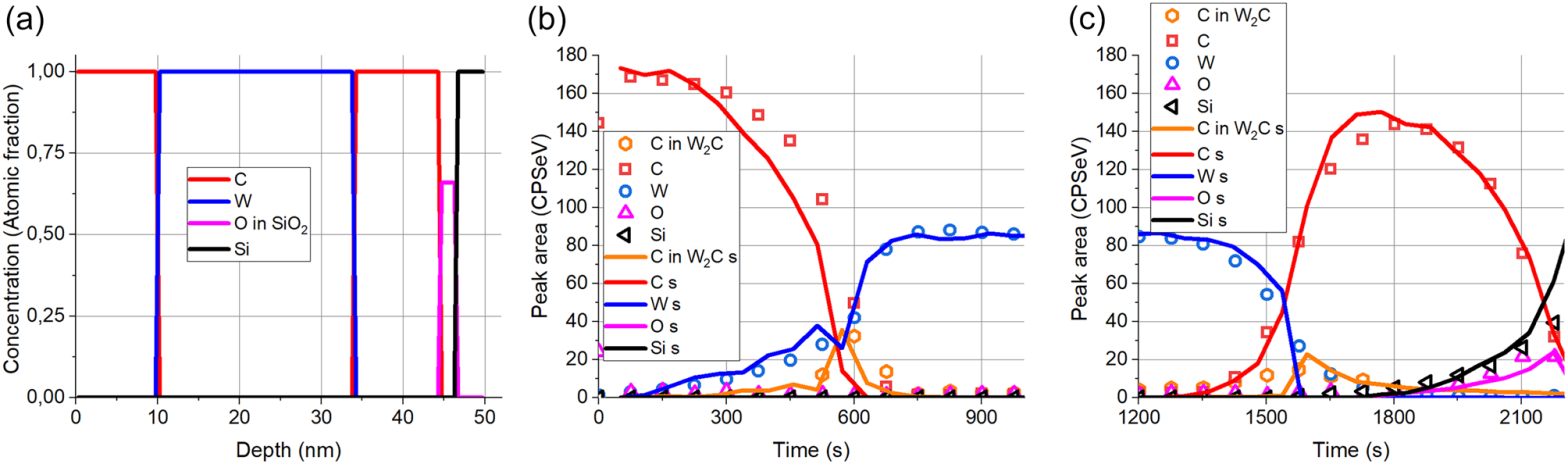
The measured and simulated XPS depth profiles on pristine sample. (**a**) Initial structure. Depth profiles (**b**) the C/W interface, (**c**) W/C interface; (symbols) measurement, (lines) simulation.

It is known that the medium energy ion–solid interaction in the case of C/W multilayers results in intermixing and compound formation^17,20^. We have also shown, rather surprisingly, that ion bombardment with energy as low as 1 keV also results in tungsten carbide formation^16^. Thus, it also follows that the sputter removal process (Ar^+^ energy is 0.5 keV) might be blamed for the appearance of the W_2_C. Accepting this we will simulate the sputter removal process applying the TRIDYN code (using the experimental sputtering conditions of Ar^+^, 500 eV 45°) which accounts for the intermixing and the model for compound formation^20^. The initial structure for the simulation is same as before. The results for the interface regions are shown in Fig. 6a,b.

Figure 6a shows the initial ideal structure of the pristine sample based on the XTEM image. This structure is the input for the simulation. In the case of the C/W interface (see Fig. 6b) the agreement between the measured and simulated profiles is excellent; it clearly shows the drastically different shapes of C lines originate from the graphite layer and that from the W_2_C produced by the sputter removal process. In case of the W/C interface (Fig. 6c) the agreement of the depth profiles of the elements are again excellent, but in the case of the sputtering induced W_2_C there is some deviation; the as measured profile of the W_2_C is broader the expected (as previously in Fig. 5a). This happens most likely because of the roughening of the interface due to the unfavorable sputtering conditions. Anyhow this result exhibits that both the distortions due to the IMFP and the sputter removal processes are correctly described by our models in most part, and shows how the initial structure of the sample results in the XPS depth profile shown in Fig. 3.

This protocol will be applied for the simulation of the XPS depth profiles obtained from samples of unknown thin layer systems.

### Medium energy irradiated samples

In this section we will report on the application of our trial and error protocol for “real” problem. The pristine sample has been irradiated by medium energy ions of various kinds and energies to produce tungsten carbide rich nano-layers at room temperature, which can be a used as corrosion protection and/or high wear resistance layers. It is evident that their structure, chemistry is to be revealed before further applications.

It has been shown above that even at Ar^+^ bombardment of 0.5 keV, used for depth profiling, intermixing and compound formation happens (producing the same W_2_C, which is produced by the medium energy irradiation) which distorts the measured depth profile, and which should be corrected.

Artifact production mainly occurs if there are pure C/pure W and/or pure W/pure C interfaces, so it is only disturbing at the beginning of the tungsten carbide production. It is favorable that the artifact production is well visible in case of XPS depth profiling. This is due to the relatively high IMFP of the excited photoelectrons. If the to be depth profiled sample contains tungsten carbide and we measure the C in W_2_C bound photoelectron (C1s) signal then due to the high IMFP (it is in pure C and W, 3.3 nm 1.7 nm, resp.) in the case of C/W interface a very broad, while in the case of W/C interface a broad peak will appear. (Note that the penetration depth of an electron is roughly 3* IMFP.) On the other hand, if the ion bombardment produces the observed tungsten carbide the corresponding C1s peak, then this peak will have a sharper appearance since the projected range of the 0.5 keV Ar^+^ ions is in the range of 1.6 ± 0.5 nm. Based on the difference of the shapes one can distinguish between the artifact and the already present tungsten carbides. Anyhow our evaluation protocol account for this problem, which will be demonstrated for the case of the sample irradiated by 0.1E16 Xe^+^/cm^2^ of 120 keV.

Making the initial guess of the irradiated sample one should remember that the medium energy irradiation produces tungsten carbide. This carbide growth from the interfaces by means of a quasi-diffusional process and thus, their initial structure will be approximated by erfc functions.

#### Sample irradiated by 0.1E16 Xe^+^/cm^2^ of 120 keV

**Figure 7:**
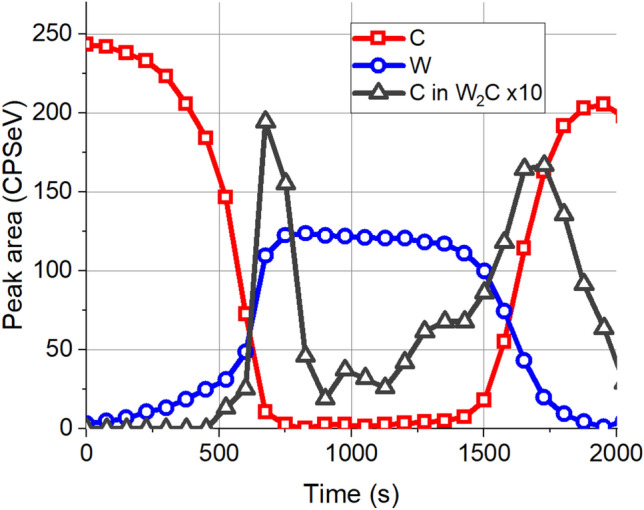
The as measured XPS depth profile obtained on sample irradiated by 0.1E16 Xe^+^/cm^2^ of 120 keV. The C 1s peak (283.6 eV) which is in carbide form signed as C in W_2_C is multiplied by 10 for better visibility.

Previous studies^20^ and simulations show that this slight irradiation causes a modest tungsten carbide production and mainly in the region of the W/C interface. The as measured XPS depth profile of sample irradiated by 0.1E16 Xe^+^/cm^2^ of 120 keV is shown in Fig. 7.

**Figure 8:**
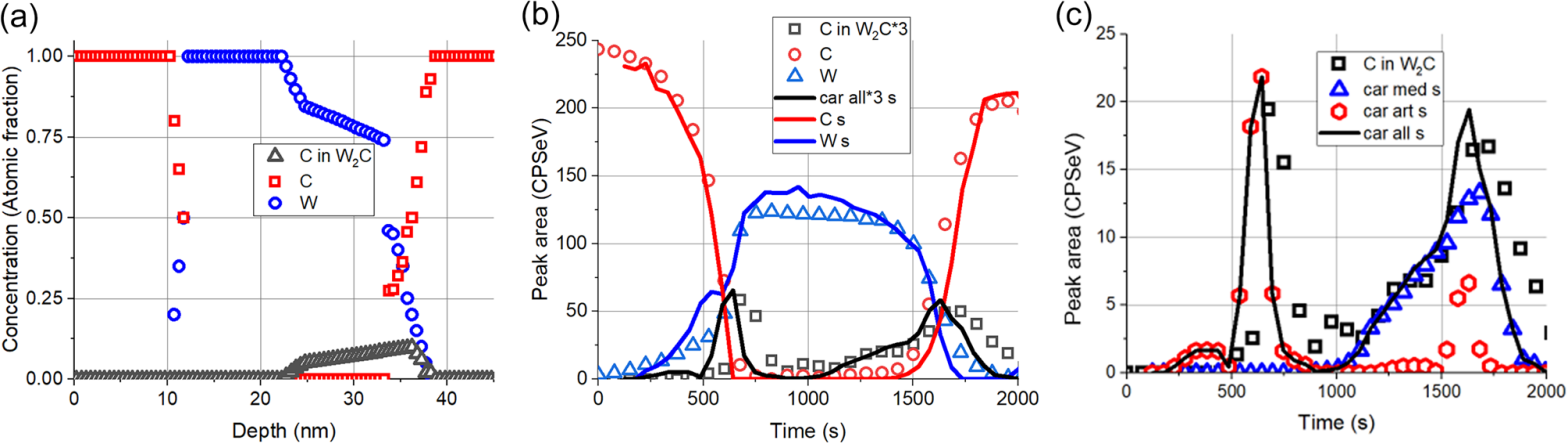
The comparison of the as measured and simulated in-depth concentration distributions of the sample after irradiation by 0.1E16 Xe^+^/cm^2^ of 120 keV. (**a**) Assumed initial concentration distributions; C, W and C in W_2_C stands for graphite, metallic W and C in W_2_C bound (produced medium energy irradiation), respectively, (**b**) comparison of the simulated (lines) and measured (symbols) depth profiles of C (graphite), W (metal) and C in W_2_C bound, (**c**) comparison of the simulated depth profiles of C in variously produced W_2_C phases with the measured C in W_2_C Car med s, car art s and car all s stand for simulated depth profiles of carbides made by the medium energy irradiation, induced by the ions used for depth profiling (artefact) and the sum of the two previous, resp.

The as measured depth profile similarly to the previous one shows two so called intermixed regions and broad transition regions. The carbon region of the XPS spectrum could be decomposed into two peaks as graphitic C (284.8 eV) and C in W_2_C carbide (283.6 eV) accordingly we have two carbon profiles one of them is the C in W_2_C signed as C in W_2_C, while the other is the graphitic C signed as C. Both the ion irradiation and ion bombardment used for depth profiling produce W_2_C, the latter is an artefact. It is evident that for determining the W_2_C produced by ion irradiation the artefact W_2_C is to be removed. This is far not easy procedure. It should be mentioned that C in W_2_C profile shows two distinct regions; a sharp feature at the C/W interface and a much broader one at the W/C interface. As we have learnt previously the sharp feature is most likely due to the artefact, while the broad transition produced by an already present W_2_C and the “intermixing” is due to the high IMFP. On the other hand, in the simulation we can easily distinguish between the two carbides; this will be shown in the following. Based on the two distinct shapes observed in the as measured profile the initial guess for the structure of the irradiated sample (which is used in the calculation of the simulated depth profiles) is the following: C/W interface is free of initial carbide, while on the W/C interface there is carbide (produced by the medium energy irradiation; its C content in the figures signed by C car and some pure W/C region. It should be emphasized that the total number of C and W atoms in the test layer system is the same as in the pristine sample except the slight C loss due to the high energy irradiation. This C loss is accounted for by changing the thickness of the first C layer. The best agreement between the simulated and the as measured depth profile was reached by choosing the initial concentration distributions shown in Fig. 8a.

Figure 8b demonstrates the measured (symbols) and simulated (lines) depth profiles, for better visibility the carbide profiles are multiplied by 3. It can be seen that the profiles are in good agreement, therefore one should accept that the assumed initial concentration distributions (Fig. 8a) are correct. Based on the simulation one can also conclude that the broad interfaces and seemingly “intermixed” regions are the consequence of the relatively high IMFP of the photoelectrons. On the other hand, the sharp feature of the C in W_2_C line at the C/W interface is an artefact (due to the ion bombardment used for depth profiling). This is detailed in Fig. 8c. The figure shows separately the simulated W_2_C carbides produced by the medium energy ion bombardment (car med s) and that produced by the ion bombardment used for depth profiling (car art s) which is an artefact. Car all s is the sum of the two previous. This figure also proves that the artefact signal is the highest in case of pure interfaces^16^.

#### Sample irradiated by 0.25E16 Xe^+^/cm^2^ of 120 keV

**Figure 9:**
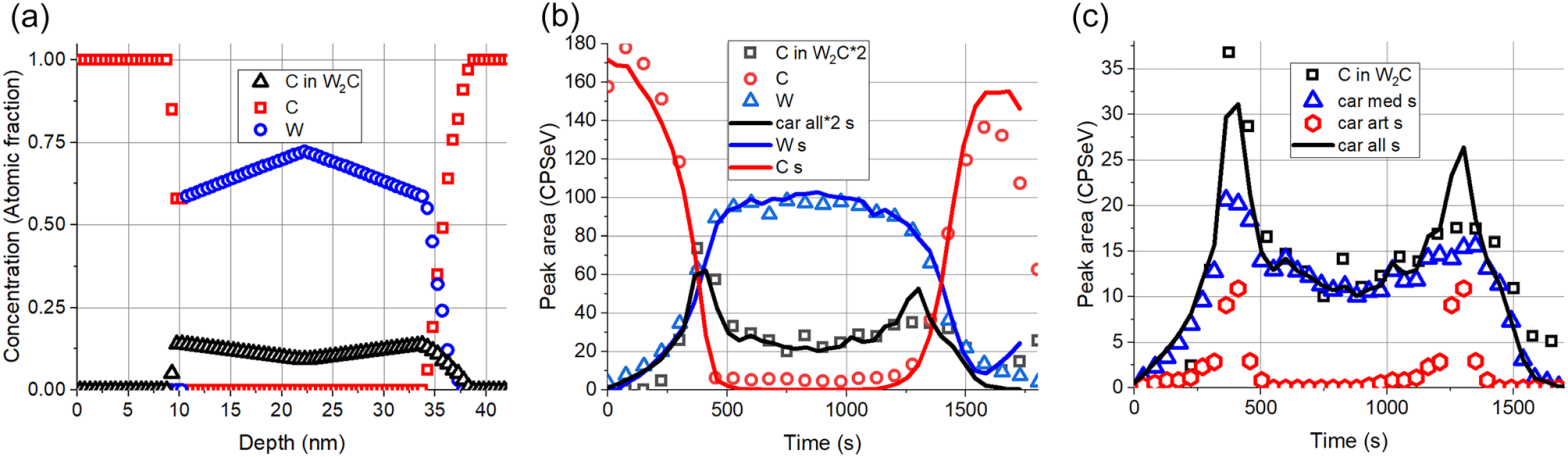
The same as Fig. 8 but after irradiation of 0.25E16 Xe^+^/cm^2^ of 120 keV.

Figure 9 shows the proposed initial structure (Fig. 9a) and the comparison of the measured and simulated depth profiles (Fig. 9b) for the sample after 0.25E16 Xe^+^/cm^2^ of 120 keV irradiation; for better visibility the low intensity carbide profiles are shown separately (Fig. 9c).

Concerning the main components, the conclusion is the same as before. On the other hand, the simulated depth profile of the C in W_2_C shows nice agreement at the C/W interface while at the W/C interface the agreement is not so good. The deviation is due to the too much higher artefact value. This suggest that the assumed initial carbide distribution covers higher part of the W/C interface than that the assumed one or as in the case of the pristine sample bombardment induced roughening occurs.

#### Sample irradiated by 1E16 Ar^+^/cm^2^ of 40 keV

**Figure 10:**
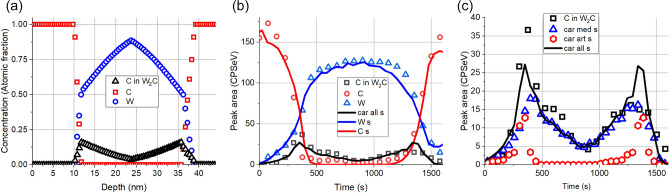
The same as Fig. 8 but after irradiation by 1E16 Ar^+^/cm^2^ of 40 keV.

Figure 10 depicts the proposed initial structure (Fig. 10a) and the comparison of the measured and simulated depth profiles (Fig. 10b) for the sample after 1E16 Ar^+^/cm^2^ of 40 keV irradiation. The carbide profiles with detailed simulation results (Fig. 10c) are also provided separately.

At this irradiation the shape of the C in W_2_C bound profile is different from the previous ones having a nearly zero value in the middle of the W layer. The simulated profiles nicely agree with the corresponding as measured ones for the main components but in the case of the C in W_2_C bound profile the agreement is reasonable and poor at the C/W and W/C interfaces, respectively and good at the other region. The deviation at the W/C interface might be explained as previously. At the C/W interface it seems that the undisturbed C/W interface is larger by 10–20% than that assumed by the amount of the carbide produced by the medium energy ions. This problem might be connected with the sharp change of the produced carbide, see Fig. 10a, in the C matrix.

In summary, we conclude using our method the initial concentration distributions of the XPS depth profiles samples could be estimated even when the as received XPS depth profiles were seriously distorted because of the high IMFP of the photoelectrons and damages introduced by the sputter removal used for the depth profiling. The results also emphasize that the routine evaluation of these measurement should not be attempted, especially in cases where carbide formation is moderate. Routine evaluation may lead to physically improper conclusions, such as intermixing of the first C layer.

The very same system has been studied by AES depth profiling^20^, where due to the much lower IMFP values of the Auger electrons, the unexpected “intermixing” had not caused any problem. The tungsten carbide production due to the depth profiling appeared similarly; anyhow the total artefact production was much lower in the case of AES depth profiling. On the other hand, the distinction of C Auger peak emitted from WC and W_2_C because of the more complex Auger process is far not straightforward, thus the identification of the carbide produced by the medium energy ion bombardment is uncertain. Thus, if one need to identify the type of carbide, XPS depth profiling is to be used even if the quality of the depth profiles is poorer than that in the case of AES depth profiling. Summarizing the proposed evaluation method can be used safely for evaluating the XPS depth profiles of complex systems.

## Conclusions

We have studied the applicability of XPS depth profiling in an unfavorable case, the C/W multilayer system, where the feature to be studied is thin, compared to the IMFP of the used photoelectrons, and sensitive to the ion bombardment used for depth profiling. Accordingly, the as measured depth profiles were strongly distorted, and routine evaluation resulted in an erroneous result for the initial structure. We proposed a trial-and-error protocol for deriving the initial concentration distributions from the distorted XPS depth profile. The problem arising from the relatively high IMFP values was handled by simulating the XP signal, considering its attenuation in matrices of varying composition. The effect of the ion bombardment was accounted for by using TRIDYN simulation. We have demonstrated for a sample of well-known structure that the method works properly, then the same protocol was applied for the study of the ion beam mixing and carbide formation in the case of medium energy (40 keV Ar^+^, 120 keV Xe^+^) ion irradiation of a C/W multilayer system. The study showed that the irradiation produced W_2_C instead of the previously assumed WC. This work demonstrates that despite of the high IMFP of the analyzed photoelectrons and artefact production due to the sputter removal process, XPS depth profiling can be still a powerful tool for analyzing the composition of nano-multilayer films.

### Supplementary Information

Supplementary Figures.

## Data Availability

Data will be available upon reasonable request from the authors (menyhard.miklos@ek.hun-ren.hu and racz.adel@ek.hun-ren.hu).
