# Supplementary material for: XPS depth profiling of nano-layers by a novel trial-and-error evaluation procedure
Source: Sci Rep. 2024 Aug 9;14:18497. doi: 10.1038/s41598-024-69495-0 (PMC11316073; doi:10.1038/s41598-024-69495-0)
Supplement: Supplementary file 1 — Supplementary Figures. [file 41598_2024_69495_MOESM1_ESM.pdf]

Supporting information

XPS depth profiling of nano-layers by a novel trial-and-error evaluation procedure

A.S. Racz†*, *M. Menyhard*†*

† Institute for Technical Physics and Materials Science, HUN-REN Centre for Energy Research, Konkoly Thege M. út 29-33, H-1121 Budapest, Hungary

*Corresponding authors: menyhard.miklos@ek.hun-ren.hu, racz.adel@ek.hun-ren.hu

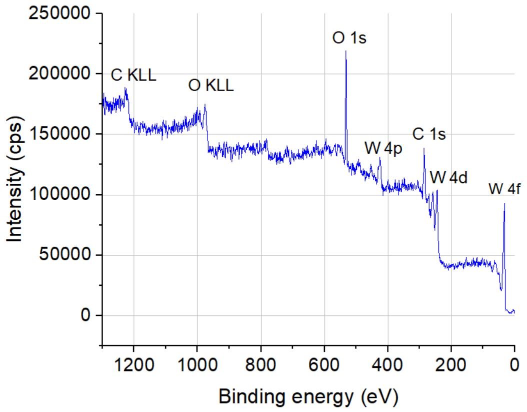

Fig. S1 Survey spectrum for the cermet sample.

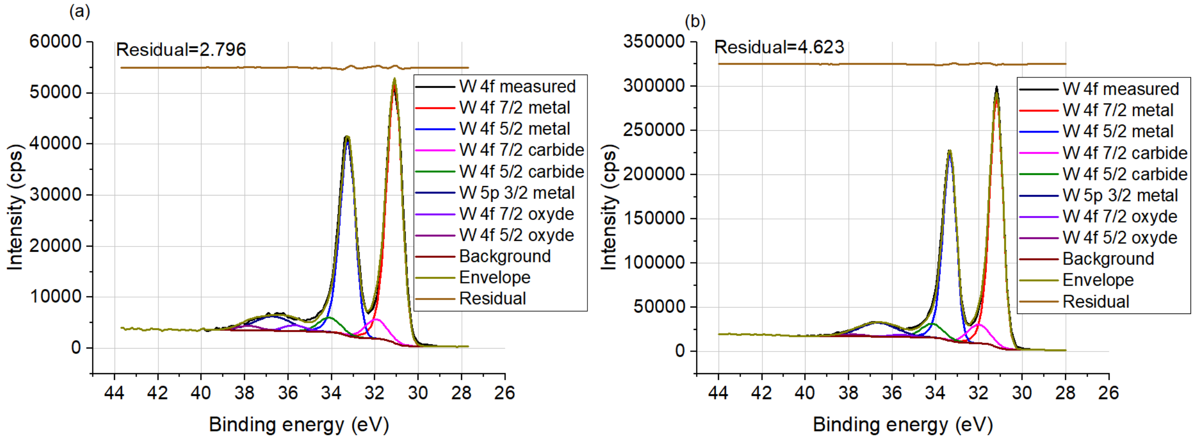

Fig. S2 The W (4f) XP spectra of the a. cermet and b. pristine sample.

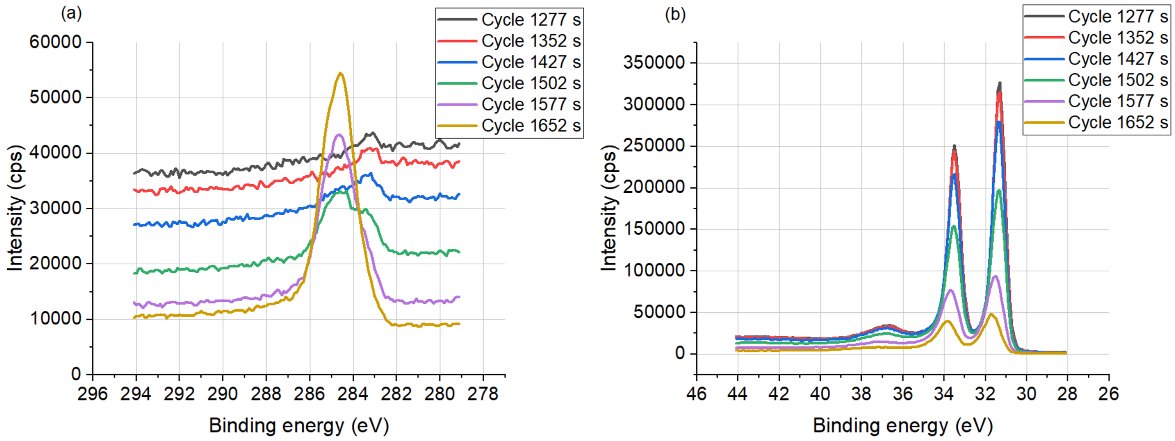

Fig. S3 a. C (1s) and b. W (4f) XP spectra for 6 consecutive sputtering steps for the pristine sample.

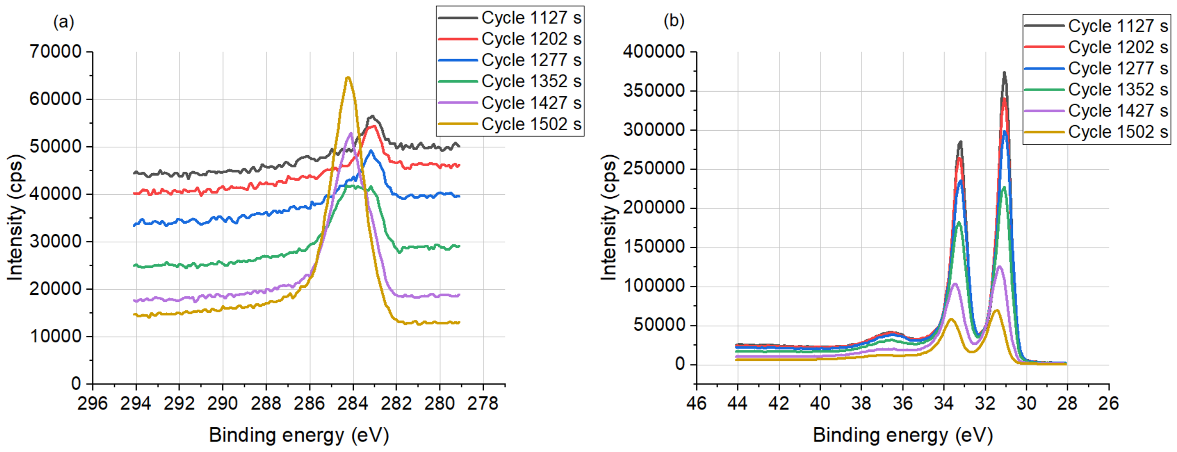

Fig.S4 a. C(1s) and b. W (4f) XP spectra for 6 consecutive sputtering steps for 40 keV 1E16Ar^{+}/cm^{2} irradiated sample.
